# Supplementary material for: Health-Related Quality of Life of Tuberculosis Patients during the COVID-19 Pandemic in Conakry, Guinea: A Mixed Methods Study
Source: Trop Med Infect Dis. 2022 Sep 2;7(9):224. doi: 10.3390/tropicalmed7090224 (PMC9506107; doi:10.3390/tropicalmed7090224)
Supplement: Supplementary file 1 [file tropicalmed-07-00224-s001.zip › tropicalmed-1852668-supplementary.pdf]

Tropical Medicine and Infectious Disease

MDPI

Supplementary Material

Table S1. Univariate analysis between dependant variables and the characteristic of the patients.

	Stress p.trend [1]	Anxiety depression p.trend [1]	Pain Discomfort p.trend [1]	Selfcare p.trend [1]	Usual activities p.trend [1]	Mobility p.trend [1]
age	0.256	0.120	0.017	0.048	<0.001	0.001
Gender	0.575	0.926	0.865	0.556	0.834	0.846
Matrimonial status	0.210	0.627	0.136	0.137	0.686	0.325
residence	0.116	0.099	0.174	0.381	0.687	0.422
Education	0.391	0.027	0.073	0.963	0.008	0.153
Occupation	0.341	0.885	0.333	0.703	0.206	0.638
Household number/size	<0.001	<0.001	0.005	0.063	0.069	0.005
Tuberculosis type	0.924	0.155	0.179	0.183	0.700	0.408
Disease localisation	0.008	0.344	0.517	0.322	0.034	0.045
Treatment history	0.656	0.347	0.449	0.622	0.041	0.148
TB diagnostic	0.005	0.113	0.067	0.227	0.072	0.710
Treatment type	0.852	0.198	0.225	0.324	0.861	0.268
Type multi-drug treatment	0.881	0.267	0.072	0.379	0.761	0.326
Anti-retroviral treatment	0.056	0.001	0.007	0.768	0.063	0.071
Distance site Patient residence	0.001	<0.001	0.182	0.025	0.185	0.313
Length time for drug procurement (in days)	0.004	<0.001	0.129	0.242	0.009	0.004
Treatment initiation	0.383	0.430	0.700	0.991	0.975	0.460
Duration of treatment	0.381	0.202	0.276	0.199	0.019	0.103

[1] p trend computed with the levels (no problem, moderate, serious) for each dependent variable.
